# Sharing Responsibility for Clathrin Coat Assembly

**DOI:** 10.1371/journal.pbio.0040301

**Published:** 2006-08-15

**Authors:** Liza Gross

Membranes protect cells from extracellular insults, but in so doing also block entry to nutrients and other essential molecules. One way cells circumvent this problem is by selectively binding such molecules to receptors on the membrane, then pulling the whole lot into the cell and packaging them into vesicles. Clathrin molecules—three-pronged pinwheel-shaped proteins—form an elaborate lattice coat around the vesicles, which ultimately bud off from the membrane and transport their cargo to their cellular destination.

This highly complex process, called clathrin-mediated endocytosis, requires a constellation of accessory proteins that interact with key protein hubs. Vesicle formation has traditionally been described as a linear process with the core proteins being clathrin and adaptor protein (AP) complexes. In a previous paper, Harvey McMahon and colleagues suggested that the process can be viewed as a network of protein interactions with clathrin and APs forming the two main hubs of the network. In a new study, Eva Schmid, Marijn Ford, McMahon, and colleagues use an impressive array of tools—biophysical, biochemical, structural, and cell biological—to shed light on the network dynamics of this “endocytic interactome.” APs orchestrate the process of cargo recruitment and assembly of the nascent vesicle and are the first hub of the endocytic network. They found that clathrin takes over from adaptors as a hub as clathrin assembles into a coat. This shift requires collaboration between the hubs, which operate within a dynamic network that performs multiple tasks simultaneously.

Of four AP complexes involved in cellular transport, AP2 figures mostly in plasma membrane endocytosis. The AP2 structure has long been likened to Mickey Mouse, with the four-subunit core representing Mickey’s body and the two flanking appendages forming his ears, but mounting evidence suggests the British children’s book character Mr. Tickle—a circular blob with gangly, elastic arms and little hands—may be a more apt comparison. Mr. Tickle’s body is the core, his arms are the two flexible hinge domains, and his hands are the two appendages, β-appendage and α-appendage. Whichever character you prefer, the core anchors the complex to the membrane and interacts with cargo molecules, and the appendages recruit accessory proteins for vesicle formation.

In their previous study, McMahon and colleagues found that α-appendages have two distinct interaction sites, allowing for clustered adaptor proteins to interact with many accessory proteins simultaneously. The AP2 α-appendage becomes a hub for protein interactions only in the initial stages of assembly. In this study, they focused on the β-appendage.

First, Schmid et al. determined the interaction partners of both appendages by removing the bound partners from cell extracts then analyzing them with mass spectrometry. They found a number of previously unidentified interaction partners for the β-appendage (and a few more for the α-appendage). Some interact only with the β-appendage, but many also interact with the α-appendage.

To understand the molecular details of the interactions, the researchers mutated key regions of the β-appendage interaction sites (the β-appendage also has a top and side site) then assessed the impact on their binding partners. They found that the top site mediates most interactions for the α-appendage and the side site does the same for the β-appendage. With this setup, accessory proteins that bind to the α-appendage’s top site can also bind to the β-appendage’s side site, leaving the appendages’ other sites free to interact with still more proteins. Interactors can bind to multiple appendages, allowing APs to serve as scaffolds for protein assembly. These results do not fully explain why two appendages exist, the researchers acknowledge, but because the same proteins interact with the top and side sites, it’s likely that the appendages collaborate to mediate these interactions.

Clathrin coat formation, Schmid et al. propose, is an outgrowth of increasingly stable interactions among a shifting network of proteins. Rapidly shifting interactions between isolated proteins give rise to coordinated, dynamic interactions between a network of proteins centered around the membrane, then to increasingly stable interactions as the coat assembles. The presence of both activated cargo receptors and lipid signaling molecules (phosphoinositides) in the membrane trigger the accumulation of adaptor complexes, which rapidly stabilize with the help of accessory proteins with multiple sites for AP2 appendage interactions. The accessory proteins recruit clathrin, which interacts with β-appendages and displaces accessory proteins as it accumulates and self-assembles during coat formation. Accessory proteins that interact only with appendages are shunted to the side, where clathrin polymers have not yet formed, while accessory proteins that can interact with clathrin are maintained. Having demonstrated the power of using a multidisciplinary approach to study the endocytic interactome, the researchers believe that the principles uncovered will apply to other protein networks.

## 

**Figure:**
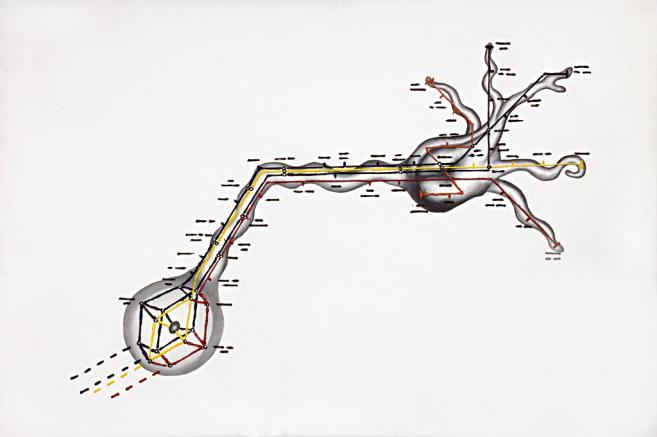
Painting by Alex McCuish (Hills Road Sixth Form College, Cambridge, United Kingdom) illustrating the protein interaction network underlying nerve function

